# Tuberculosis treatment outcomes of diabetic and non-diabetic TB/HIV co-infected patients: A nationwide observational study in Brazil

**DOI:** 10.3389/fmed.2022.972145

**Published:** 2022-09-16

**Authors:** Klauss Villalva-Serra, Beatriz Barreto-Duarte, Vanessa M. Nunes, Rodrigo C. Menezes, Moreno M. S. Rodrigues, Artur T. L. Queiroz, María B. Arriaga, Marcelo Cordeiro-Santos, Afrânio L. Kritski, Timothy R. Sterling, Mariana Araújo-Pereira, Bruno B. Andrade

**Affiliations:** ^1^Multinational Organization Network Sponsoring Translational and Epidemiological Research (MONSTER) Initiative, Salvador, Brazil; ^2^Curso de Medicina, Universidade Salvador (UNIFACS), Salvador, Brazil; ^3^Programa de Pós-Graduação em Medicina Tropical, Universidade do Estado do Amazonas, Manaus, Brazil; ^4^Laboratório de Inflamação e Biomarcadores, Instituto Gonçalo Moniz, Fundação Oswaldo Cruz, Salvador, Brazil; ^5^Programa de Pós-Graduação em Clínica Médica, Universidade Federal do Rio de Janeiro, Rio de Janeiro, Brazil; ^6^Faculdade de Medicina, Universidade Federal da Bahia, Salvador, Brazil; ^7^Grupo de Estudos em Medicina Intensiva (GEMINI), Salvador, Brazil; ^8^Laboratório de Análise e Visualização de Dados, Fundação Oswaldo Cruz, Porto Velho, Brazil; ^9^Center of Data and Knowledge Integration for Health, Instituto Gonçalo Moniz, Fundação Oswaldo Cruz, Salvador, Brazil; ^10^Instituto de Medicina Tropical Alexander Von Humboldt, Universidad Peruana Cayetano Heredia, Lima, Peru; ^11^Fundação Medicina Tropical Doutor Heitor Vieira Dourado, Manaus, Brazil; ^12^Faculdade de Medicina, Universidade Nilton Lins, Manaus, Brazil; ^13^Programa Acadêmico de Tuberculose da Faculdade de Medicina, Universidade Federal do Rio de Janeiro, Rio de Janeiro, Brazil; ^14^Division of Infectious Diseases, Department of Medicine, Vanderbilt University School of Medicine, Nashville, TN, United States; ^15^Curso de Medicina, Faculdade de Ciência e Tecnologia (UNIFTC), Salvador, Brazil

**Keywords:** HIV, tuberculosis, treatment outcome, diabetes, Antiretroviral therapy (ART)

## Abstract

**Background:**

Tuberculosis (TB) is a worldwide public health problem, especially in countries that also report high numbers of people living with HIV (PLWH) and/or diabetes mellitus (DM). However, the unique features of persons with TB-HIV-DM are incompletely understood. This study compared anti-TB treatment (ATT) outcomes of diabetic and non-diabetic TB/HIV co-infected patients.

**Methods:**

A nationwide retrospective observational investigation was performed with data from the Brazilian Tuberculosis Database System among patients reported to have TB-HIV co-infection between 2014 and 2019. This database includes all reported TB cases in Brazil. Exploratory and association analyses compared TB treatment outcomes in DM and non-DM patients. Unfavorable outcomes were defined as death, treatment failure, loss to follow-up or recurrence. Multivariable stepwise logistic regressions were used to identify the variables associated with unfavorable ATT outcomes in the TB-HIV population.

**Results:**

Of the 31,070 TB-HIV patients analyzed, 999 (3.2%) reported having DM. However, in these TB-HIV patients, DM was not associated with any unfavorable treatment outcome [adjusted Odds Ratio (aOR): 0.97, 95% CI: 0.83–1.12, *p* = 0.781]. Furthermore, DM was also not associated with any specific type of unfavorable outcome in this study. In both the TB-HIV group and the TB-HIV-DM subpopulation, use of alcohol, illicit drugs and tobacco, as well as non-white ethnicity and prior TB were all characteristics more frequently observed in persons who experienced an unfavorable ATT outcome.

**Conclusion:**

DM is not associated with unfavorable TB treatment outcomes in persons with TB-HIV, including death, treatment failure, recurrence and loss to follow up. However, consumption habits, non-white ethnicity and prior TB are all more frequently detected in those with unfavorable outcomes in both TB-HIV and TB-HIV-DM patients.

## Introduction

*Mycobacterium tuberculosis* (MTB) is the etiologic agent of tuberculosis (TB) and is estimated to infect up to one quarter of the world's population; TB is also one of the leading causes of death (1). Notably, Brazil is one of the 20 countries with highest TB-HIV burden worldwide (2).

There is an important association between TB and diabetes mellitus (DM). DM is associated with a higher risk of developing active TB, of experiencing unfavorable anti-TB treatment (ATT) outcomes, including TB recurrence (3–5). However, although HIV and DM have a significant impact on TB, there are few data available on the co-occurrence of these three diseases (TB-HIV-DM).

Patients living with TB and HIV have higher mortality and morbidity rates compared to persons with only HIV (6). Currently, of the 10 million people globally who developed TB in 2019, 8.2% were persons living with HIV (PLWH) (1). There were 215,000 TB-HIV patients globally who died in 2020 (1). Additionally, when such patients have additional conditions such as DM, smoking, alcoholism or malnutrition, the occurrence of poor outcomes of TB-HIV co-infection become even more pronounced (7).

Several studies have demonstrated that TB patients with either HIV or DM have a worse prognosis compared to patients without these co-morbidities, especially when other social or epidemiological factors are present (3, 5, 8, 9). Furthermore, a previous cross-sectional study conducted by our group has demonstrated that TB-HIV patients with DM have higher positive smear and abnormal x-ray rates, as well as more frequent use of alcohol and tobacco when compared to those without DM (10). However, to the best of our knowledge, there have not been any prior studies specifically assessing the effects of concomitant HIV and DM on ATT outcome. The present study utilized the Brazilian Tuberculosis Database System (SINAN-TB), with all TB cases reported in Brazil from 2014 to 2019, to investigate the factors associated with TB treatment outcomes among persons with TB-HIV, as well as the effects of DM in this population.

## Materials and methods

### Ethics statement

All data accessed in this study was obtained from a publicly available platform, pre-processed by the Brazilian government's Ministry of Health. This program verifies its data in matters of consistency, duplicate registration, and completeness, following the instructions set by Resolution Number 466/12 on Research Ethics of the National Health Council, Brazil (11).

### Overall study design

We performed a retrospective observational study, using data from the Information System for Notifiable Diseases—Tuberculosis database (SINAN-TB), from 2009 to 2019, to characterize TB treatment outcomes in the Brazilian TB-HIV population, with and without DM. The SINAN-TB database is maintained, verified, and updated by the Brazilian Ministry of Health. We have removed pregnant women, homeless and incarcerate patients of this database. We also removed all patients with lack in diabetes status.

### Data collection

Data was collected between the years of 2014 and 2019 with TB being diagnosed by bacteriology/positive culture, chest radiography or histopathology or clinical manifestations, following criteria established by the Brazilian Ministry of Health, further detailed in the Manual of Recommendations for TB Control in Brazil (12). Additionally, SINAN-TB obtains data concerning social and clinical characteristics of all notified TB cases in the Brazilian population.

### Outcome definition

In our study, an unfavorable outcome was the composite of death, treatment failure, loss to follow-up or recurrence; favorable outcome was defined as cure (clinical or bacteriological). The definitions for both groups regarding clinical and bacteriological cure, treatment failure, loss to follow-up, recurrence and death were defined following the instructions in the Manual of Recommendations for the Control of TB of Brazil (12). The outcomes “unknown,” “ongoing treatment” and “transferred out” were not considered for the analysis of TB treatment outcomes. The outcome definitions are shown in Supplementary Table 1.

### Study definitions

In this paper, we defined the variables utilized in the following manner: diabetes mellitus: self-reported diagnosis of DM, alcohol consumption: any past or current consumption of alcohol; tobacco use: past or current smoking of tobacco; illicit drug use: past or current illicit drug use (marijuana, cocaine, heroin or crack); non-white: the following self-reported races or ethnicities: Asian, Black, *Pardo* and Indigenous; type of TB: clinical form of TB regarding the disease's location, grouped into two categories: pulmonary TB (PTB) and Non-PTB, which included both extrapulmonary TB (EPTB) and disseminated TB (PTB + EPTB); supervised treatment: directly observed therapy (DOT); prior-TB: previous TB history; education stages: patient's education was self-reported and divided into two groups, those without a completed high school degree and those with either a high school diploma, with some college experience or a completed college degree; age groups: patient age was divided into three categories, according to the interquartile-range of the age of all patients during the study period: <40, 40–56, and > 56 years old.

### Statistical analysis

Social and clinical characteristics classified as categorical variables were presented as percentages (%) and compared using the Pearson's chi-squared test (χ^2^). Continuous variables were represented by interquartile range (IQR) and medians, and the Mann-Whitney *U*-test was used for comparisons of groups. *p* values <0.05 were considered statistically significant. We also employed second-generation *p*-values (*p*δ) as described previously (13) to examine statistically relevant associations in this large dataset. In addition to *p*δ, we also calculated the delta-gap (**Δ**), which was designated as the distance between intervals of δ units. The calculation of the delta-gap value was related to the differences in distribution between the results of each group, considering that in cases of *p*-value <0.05, a delta value of was considered statistically significant. Additionally, the effect size of the model grows as the delta value increases.

We performed two binomial logistic regression models with a stepwise method (ENTER), selecting all variables that were included in the univariable analysis, to assess associations between the clinical and social characteristics with unfavorable treatment outcomes in TB-HIV patients, utilizing DM as a predictor variable, as well as evaluating its association with specific unfavorable outcomes.

## Results

### Treatment outcomes of TB-HIV co-infected patients

A total of 31,070 patients with TB-HIV co-infection in Brazil were reported to SINAN-TB between 2014 and 2019, with 999 (3.2%) of these patients self-reporting a diagnosis of DM. Data comparing clinical and epidemiological characteristics that are present in the TB-HIV-DM and TB-HIV groups can be found in Supplementary Table 2. In this study, we analyzed these subpopulations regarding different ATT outcomes.

Regarding the differences in treatment outcomes between these two groups, we found that those without DM had higher frequency of a favorable outcome than individuals with TB-HIV-DM (TB-HIV: 45.0%, TB-HIV-DM: 43.9%, *p* < 0.001). Additionally, individuals in the TB-HIV-DM group also presented with higher mortality (TB-HIV: 19.8%, TB-HIV-DM: 24.0%) and treatment failure (TB-HIV: 3.5%; TB-HIV-DM: 4.4%), while loss to follow up (TB-HIV: 14.3%; TB-HIV-DM: 14.2%) and recurrence (TB-HIV: 17.5%; TB-HIV-DM: 13.4%) rates were more prevalent in the non-DM group (χ^2^ *p* < 0.001, *p*δ = 0, Δ = 0.23) (Table 1). However, when comparing the composite outcome (favorable vs. unfavorable), there were no differences founded between groups (*p* = 0.514) (Table 1).

**Table 1:** Comparison of treatment outcomes between TB-HIV and TB-HIV-DM population.

**Characteristics**	**All** ** (*n* = 31,070)**	**DM** ** (*n* = 999)**	**Non-DM** ** (*n* = 30,071)**	***p*-Value**	***pδ*-Value**
**Outcome description**, ***n*** **(%):**				**<0.001**	ns
Cure	13,967 (44.9%)	439 (43.9%)	13,528 (45.0%)		
Death	6,191 (19.9%)	240 (24.0%)	5,951 (19.8%)		
Failure	1,088 (3.50%)	44 (4.4%)	1,044 (3.5%)		
Lost to follow up	4,414 (14.2%)	142 (14.2%)	4,299 (14.3%)		
Recurrence	5,383 (17.3%)	134 (13.4%)	5,249 (17.5%)		
**Outcome**, ***n*** **(%)**					
Favorable	13,967 (45.0%)	439 (43.9%)	13,528 (45.0%)	0.514	ns
Unfavorable	17,076 (55.0%)	560 (56.1%)	17,542 (56.4%)		

Data represents frequency (%). Categorical variables were compared using Pearson's chi-square test. Definition of outcome description can be found at Supplementary Table 1. DM, diabetes mellitus; ns, not significant; pδ-value, second-generation p-value; Δ, delta-gap. Bold values indicate statistically significant p-values.

When differentiating TB-HIV patients concerning their classification into the favorable or unfavorable outcome groups, those with unfavorable outcomes had higher frequencies of biological and social factors such as non-white ethnicity (69.4%, χ^2^ *p* < 0.001, *p*δ = 0, Δ = 0.60), alcohol consumption (25.4%, χ^2^ *p* < 0.001, *p*δ = 0, Δ = 0.12), illicit drug use (24.7%, χ^2^ *p* < 0.001, *p*δ = 0, Δ = 0.15) and smoking tobacco (24.4%, χ^2^ *p* < 0.001, *p*δ = 0, Δ = 0.07). In addition, clinical variables such as history of prior TB (34.0%, χ^2^ *p* < 0.001, *p*δ = 0, Δ = 0.23) (Table 2) were also more common in persons who experienced unfavorable outcomes. In contrast, no statistically significant variables were more frequently observed in the favorable outcome group. Additionally, DM did not find a statistically higher frequency in either group of treatment outcomes in these TB-HIV patients (χ^2^ *p* = 0.536, *p*δ = 0.33) (Table 2).

**Table 2:** Characterization of treatment outcomes in the TB-HIV population.

**Characteristics**	**All** ** (*n* = 31,070)**	**Favorable** ** (*n* = 13,967)**	**Unfavorable** ** (*n* = 17,103)**	***p*-Value**	***pδ*-Value**
Male, *n* (%):	21,801 (70.2%)	10,079 (72.2%)	11,722 (68.5%)	**<0.001**	1
**Age categories**, ***n*** **(%):**				**0.04**	0.09
Lower than 40 years	16,929 (54.5%)	7,540 (54.0%)	9,389 (54.9%)		
Between 40–56 years	11,846 (38.1%)	5,402 (38.7%)	6,444 (37.7%)		
Higher than 56 years	2,295 (7.4%)	1,025 (7.3%)	1,270 (7.4%)		
Non-white, *n* (%):	20,931 (67.4%)	9,112 (65.3%)	11,860 (69.4%)	**<0.001**	**0 (Δ** **=** **0.60)**
**Educational stages**, ***n*** **(%):**				**<0.001**	0.67
Less than HS diploma	3,069 (10.8%)	1,375 (10.7%)	1,694 (10.8%)		
More than HS diploma	25,396 (89.2%)	11,440 (89.3%)	13,956 (89.2%)		
HIV, *n (%):*	31,070 (100%)	13,967 (100%)	17,103 (100%)	ns	ns
Alcohol consumption, *n* (%):	6,733 (22.2%)	2,492 (18.1%)	4,241 (25.5%)	**<0.001**	**0 (Δ** **=** **0.12)**
Illicit drug use, *n* (%):	5,987 (20.4%)	2,036 (15.3%)	3,951 (24.7%)	**<0.001**	**0 (Δ** **=** **0.15)**
Tobacco use, *n* (%):	6,474 (21.9%)	2,542 (19.0%)	3,932 (24.4%)	**<0.001**	**0 (Δ** **=** **0.07)**
DM, *n* (%):	999 (3.2%)	439 (3.14%)	560 (3.27%)	0.536	0.33
Smear positive, *n* (%):	10,850 (96.4%)	5,302 (96.1%)	5,548 (96.6%)	0.118	1
Culture positive, *n* (%):	5,628 (58.1%)	2,729 (57.0%)	2,899 (59.2%)	0.026	1
Suspect chest x-ray for TB, *n* (%):	22,482 (73.7%)	10,106 (73.8%)	12,376 (73.6%)	**<0.001**	0.49
Supervised treatment, *n* (%)	3,011 (9.7%)	1,805 (12.9%)	1,206 (7.0%)	**<0.001**	1
Prior TB, *n* (%):	8,780 (28.3%)	2,958 (21.2%)	5,822 (34.0%)	**<0.001**	**0 (Δ** **=** **0.23)**
**Type of TB**, ***n*** **(%):**				0.971	0.5
PTB	20,925 (67.3%)	9,408 (67.4%)	11,517 (67.3%)		
Non-PTB	10,145 (32.7%)	4,559 (32.6%)	5,586 (32.7%)		
**Outcome description**, ***n*** **(%):**					
Cure	13,967 (44.9%)	13,967 (100%)	0 (0.00%)	**<0.001**	ns
Death	6,191 (19.9%)	0 (0.00%)	6,191 (36.2%)		
Failure	1,088 (3.50%)	0 (0.00%)	1,088 (6.36%)		
Lost to follow up	4,441 (14.2%)	0 (0.00%)	4,414 (25.8%)		
Recurrence	5,383 (17.3%)	0 (0.00%)	5,383 (31.5%)		

Data represents frequency (%). Categorical variables were compared using Pearson's chi-square test. Definition of educational stages: Self-reported education level divided into HS completion or uncompletion. Definition of alcohol consumption: Past or current any consumption of alcohol. Definition of tobacco use: Past or current smoking of tobacco. Definition of illicit drug use: Past or current illicit drug use (marijuana, cocaine, heroin or crack) Definition of non-white: The following self-reported races: Asian, Black, Pardo, and Indigenous. Definition of type of TB: Clinical form of TB regarding the disease's location. Definition of Supervised Treatment: Directly Observed Therapy (DOT). Definition of prior-TB: Previous TB history. TB, tuberculosis; PTB, pulmonary tuberculosis; Non-PTB, extrapulmonary and disseminated tuberculosis; HIV, human immunodeficiency virus; DM, diabetes mellitus; HS, high school; ns, not significant; pδ-value, second-generation p-value; Δ, delta-gap. Bold values indicate statistically significant p-values.

### Treatment outcomes in the TB-HIV-DM population

**Figure 1:**
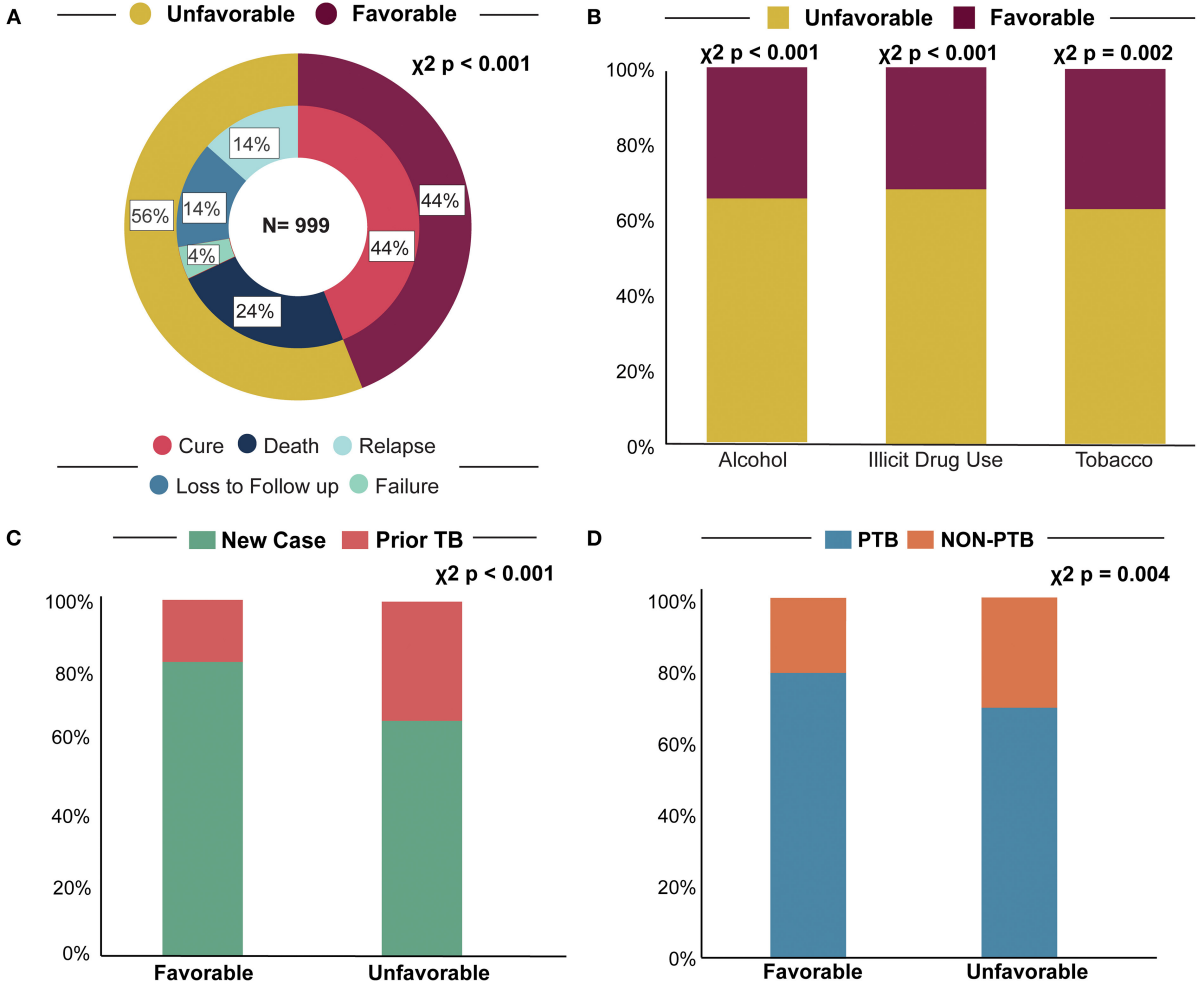
Determinant factors for treatment outcome. **(A)** Treatment outcome, most TB-HIV-DM patients had an unfavorable treatment outcome **(B)**. Consumption habits, we demonstrated that alcohol consumption, illicit drug, and tobacco usage all had higher rates of negative outcome. **(C)** TB status, we found that being a new case of TB had a protective factor against unfavorable outcomes in patients with TB-HIV-DM. **(D)** TB type, we observed that patients with PTB obtained a higher percentage of favorable outcomes, while non-PTB cases had higher rates of negative outcome. TB, tuberculosis; PTB, pulmonary tuberculosis; Non-PTB, extrapulmonary and disseminated tuberculosis.

Next, we assessed the different treatment outcomes specifically in the TB-HIV-DM population. There were 439 patients with a favorable outcome (43.9%), while 560 cases experienced unfavorable outcomes (56.1%) (Table 3). Among unfavorable outcomes, death (24.0%) was the most common cause, followed by loss to follow up (25.4%), recurrence (23.9%) and treatment failure (7.68%) (*p* < 0.001) (Table 3; Figure 1A).

**Table 3:** Characterization of treatment outcomes in the TB-HIV-DM population.

**Characteristics**	**All** ** (*n* = 999)**	**Favorable** ** (*n* = 439)**	**Unfavorable** ** (*n* = 560)**	***p*-Value**	***pδ*-Value**
Male, *n* (%):	690 (69.1%)	302 (68.8%)	388 (69.3%)	0.922	0.45
**Age categories**, ***n*** **(%)**				0.392	0.53
Lower than 40	239 (23.9%%)	97 (22.1%)	142 (25.4%)		
40 through 56	516 (51.7%%)	228 (51.9%)	288 (51.4%)		
Higher than 56	244 (24.4%%)	114 (26.0%)	130 (23.2%)		
Non-white, *n* (%):	674 (67.5%)	274 (62.4%)	400 (71.4%)	**0.003**	**0 (Δ** **=** **0.05)**
**Educational stages**, ***n*** **(%):**				0.72	0.4
Less than HS diploma	121 (13.5%)	51 (13.0%)	70 (13.5%)		
More than HS diploma	775 (86.5%)	340 (87.0%)	435 (86.4%)		
Alcohol consumption, *n* (%):	277 (29.4%)	97 (22.8%)	180 (34.9%)	**<0.001**	**0 (Δ** **=** **0.08)**
Illicit drug use, *n* (%):	172 (19.3%)	56 (13.8%)	116 (24.0%)	**<0.001**	**0 (Δ** **=** **0.06)**
Tobacco use, *n* (%):	262 (29.0%)	98 (23.8%)	164 (33.3%)	**0.002**	**0 (Δ** **=** **0.02)**
Smear positive, *n* (%):	410 (97.9%)	211 (96.8%)	199 (99.0%)	0.178	1
Culture positive, *n* (%):	198 (59.8%)	90 (57.7%)	108 (61.7%)	0.527	0.61
Suspected chest x-ray for TB, *n* (%):	741 (74.9%)	334 (77.1%)	407 (73.2%)	0.226	0.8
Supervised treatment, *n* (%)	254 (43.3%)	155 (48.6%)	99 (36.9%)	**0.006**	1
Prior TB, *n* (%):	276 (27.6%)	80 (18.2%)	196 (35.0%)	**<0.001**	**0 (Δ** **=** **0.22)**
**Type of TB**, ***n*** **(%):**				**0.004**	1
PTB	747 (74.8%)	351 (80.0%)	396 (70.7%)		
Non-PTB	252 (25.2%)	88 (20.0%)	164 (29.3%)		
**Outcome description**, ***n*** **(%):**				**<0.001**	ns
Cure	439 (43.9%)	439 (100%)	0 (0.00%)		
Death	240 (24.0%)	0 (0.00%)	240 (42.9%)		
Failure	44 (4.40%)	0 (0.00%)	44 (7.86%)		
Lost to follow up	142 (14.2%)	0 (0.00%)	142 (25.4%)		
Recurrence	134 (13.4%)	0 (0.00%)	134 (23.9%)		

Data represents frequency (%). Categorical variables were compared using Pearson's chi-square test. Definition of educational stages: Self-reported education level divided into HS completion or uncompletion. Definition of alcohol consumption: Past or current any consumption of alcohol. Definition of tobacco use: Past or current smoking of tobacco. Definition of illicit drug use: Past or current illicit drug use (marijuana, cocaine, heroin or crack) Definition of non-white: The following self-reported races: Asian, Black, Pardo, and Indigenous. Definition of type of TB: Clinical form of TB regarding the disease's location. Definition of Supervised Treatment: Directly Observed Therapy (DOT). Definition of prior-TB: Previous TB history. TB, tuberculosis; PTB, pulmonary tuberculosis; Non-PTB, extrapulmonary and disseminated tuberculosis; HS, high school; pδ-value, second-generation p-value; Δ, delta-gap. Bold values indicate statistically significant p-values.

While comparing patients who had favorable vs. unfavorable outcome, those with an unfavorable outcome had higher frequencies of social factors like presence of alcohol consumption (χ^2^ *p* < 0.001, *p*δ = 0, Δ = 0.08), illicit drug use (χ^2^ *p* < 0.001, *p*δ = 0, Δ = 0.06), and smoking tobacco (χ^2^ *p* = 0.002, *p*δ = 0, Δ = 0.02) (Table 3; Figure 1B). Additionally, non-white race (71.4%, χ^2^ *p* = 0.003, *p*δ = 0, Δ = 0.05) patients were also more prevalent among those with an unfavorable outcome (Table 3). Conversely, being a new case of TB (81.8%, χ^2^ *p* < 0.001, *p*δ = 0, Δ = 0.22) was more frequent in those with a favorable outcome (Table 3; Figure 1C). Finally, regarding type of TB, we observed that those with non-PTB form (EPTB and PTB + EPTB) had higher frequency of unfavorable outcomes (Table 3; Figure 1D).

### Treatment outcomes in the TB-HIV population without DM

We next analyzed the same treatment outcomes in a population of TB-HIV patients without DM. Curiously, most statistically significant variables were the same in both populations, including non-white ethnicity (69.1%, χ^2^ *p* < 0.001, *p*δ = 0, Δ = 0.06), alcohol consumption (25.2%, χ^2^ *p* < 0.001, *p*δ = 0, Δ = 0.08), illicit drug use (24.7%, χ^2^ *p* < 0.001, *p*δ = 0, Δ = 0.15), smoking tobacco (24.0%, χ^2^ *p* < 0.001, *p*δ = 0, Δ = 0.07), prior TB (34.0%, χ^2^ *p* < 0.001, *p*δ = 0, Δ = 0.23), while male sex, age, educational stages and smear microscopy were only significant in the non-DM population. These data are shown in detail on Table 4.

**Table 4:** Characterization of treatment outcomes in TB-HIV without DM.

**Characteristics**	**All** ** (*n* = 30,044)**	**Favorable** ** (*n* = 13,528)**	**Unfavorable** ** (*n* = 16,516)**	***p*-Value:**	***pδ*-Value**
Male, *n* (%):	21,093 (70.2%)	9,777 (72.3%)	11,316 (68.5%)	**<0.001**	1
**Age categories**, ***n*** **(%):**				0.203	0.6
Lower than 40	27,998 (93.2%)	12,617 (93.3%)	15,381 (93.1%)		
Between 40–56	158 (0.53%)	60 (0.44%)	98 (0.59%)		
Higher than 56	1,888 (6.28%)	851 (6.29%)	1,037 (6.28%)		
Non-white, *n* (%):	20,242 (67.3%)	8,819 (65.1%)	11,423 (69.1%)	**<0.001**	**0 (Δ** **=** **0.06)**
**Educational stages**, ***n*** **(%):**				0.849	0.79
Less than HS diploma	2,946 (10.7%)	1,324 (10.6 %)	1,622 (10.7%)		
More than HS diploma	24,597 (89.3%)	11,100 (89.4%)	13,479 (89.3%)		
HIV, *n (%):*	30,044 (100%)	13,528 (100%)	16,516 (100%)	ns	ns
Alcohol consumption, *n* (%):	6,450 (21.4%)	2,395 (17.9%)	4,055 (25.2%)	**<0.001**	**0 (Δ** **=** **0.08)**
Illicit drug use, *n* (%):	5,810 (19.3%)	1,980 (15.3%)	3,830 (24.7%)	**<0.001**	**0 (Δ** **=** **0.15)**
Tobacco, *n* (%):	6,212 (20.6%)	2,444 (18.8%)	3,768 (24.0%)	**<0.001**	**0 (Δ** **=** **0.07)**
Not-DM, *n* (%):	30,044 (100%)	13,528 (100%)	16,516 (100%)	**ns**	
Smear positive, *n* (%):	10,428 (34.7%)	5,091 (96.0%)	5,349 (96.5%)	0.163	1
Culture positive, *n* (%):	5,422 (18.0%)	2,639 (57.0%)	2,783 (59.1%)	0.032	1
Suspect chest x-ray, *n* (%):	21,721 (72.2%)	9,772 (73.7%)	11,949 (73.6%)	**<0.001**	0.44
Supervised treatment, *n* (%)	2,955 (9.83%)	1,771 (61.4%)	1,184 (46.5%)	**<0.001**	1
Prior TB, *n* (%):	8,946 (29.7%)	2,878 (21.2%)	5,618 (34.0%)	**<0.001**	**0 (Δ** **=** **0.23)**
**Type of TB**, ***n*** **(%):**				**<0.001**	**0.36**
PTB	20,161 (67.1%)	9,057 (66.9%)	11,104 (67.2%)		
Non-PTB	9,893 (32.9%)	4,471 (33.0%)	5,422 (32.8%)		
**Outcome description**, ***n*** **(%):**				**<0.001**	ns
Cure	13,528 (45.0%)	13,528 (100%)	0 (0.00%)		
Death	5,951 (19.8%)	0 (0.00%)	5,951 (36.0%)		
Failure	1,044 (3.47%)	0 (0.00%)	1,044 (6.32%)		
Lost to follow up	4,272 (14.2%)	0 (0.00%)	4,272 (25.8%)		
Recurrence	5,249 (17.4%)	0 (0.00%)	5,249 (31.7%)		

Data represents frequency (%). Categorical variables were compared using Pearson's chi-square test. Definition of educational stages: Self-reported education level divided into HS completion or uncompletion. Definition of alcohol consumption: Past or current any consumption of alcohol. Definition of tobacco use: Past or current smoking of tobacco. Definition of illicit drug use: Past or current illicit drug use (marijuana, cocaine, heroin or crack) Definition of non-white: The following self-reported races: Asian, Black, Pardo, and Indigenous. Definition of type of TB: Clinical form of TB regarding the disease's location. Definition of Supervised Treatment: Directly Observed Therapy (DOT). Definition of prior-TB: Previous TB history. TB, tuberculosis; PTB, pulmonary tuberculosis; Non-PTB, extrapulmonary and disseminated tuberculosis; HS, high school; ns, not significant; pδ-value, second-generation p-value; Δ, delta-gap. Bold values indicate statistically significant p-values.

### Comparison of treatment outcomes between TB-HIV and TB-HIV-DM population

**Figure 2:**
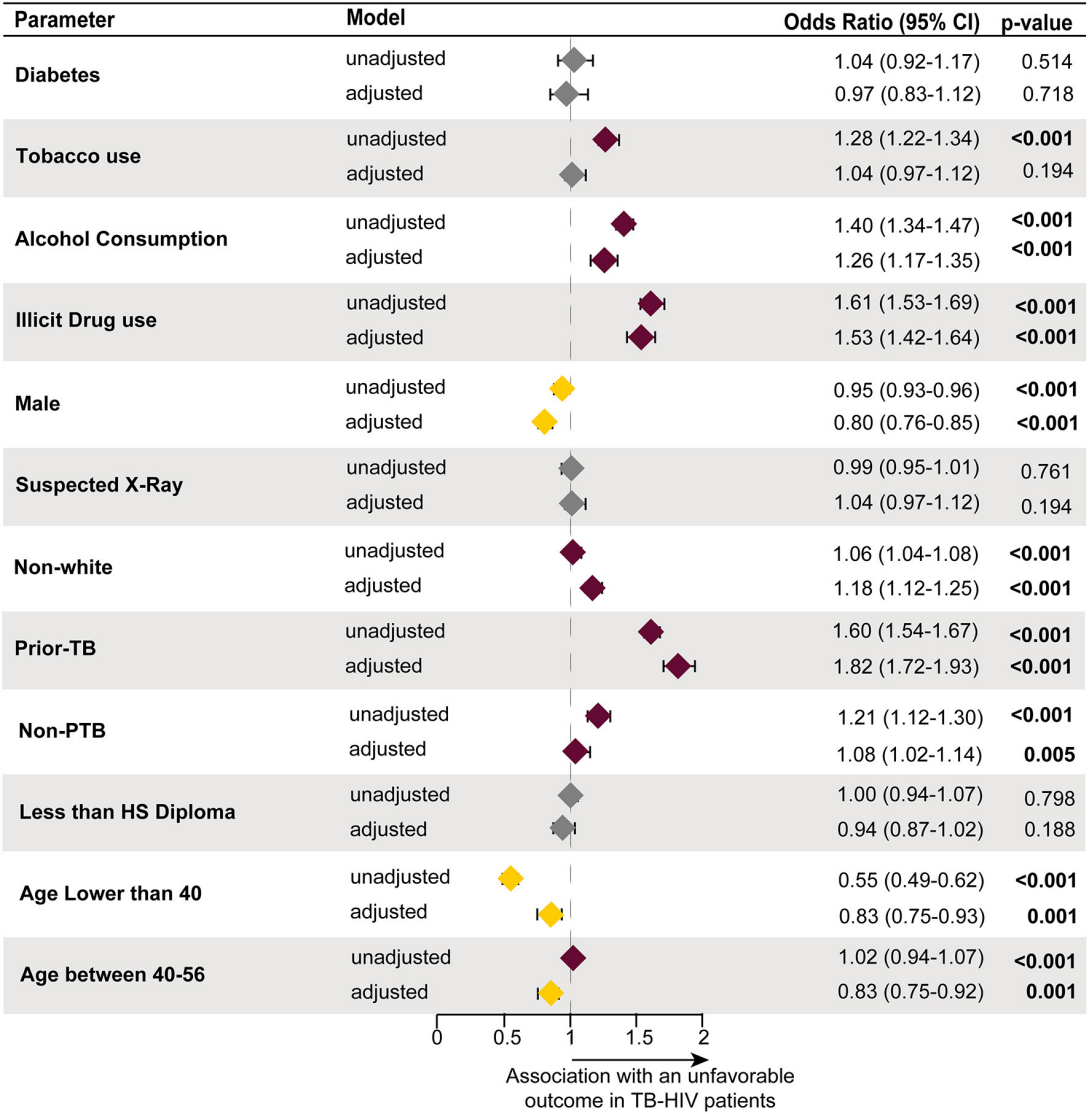
An enter method binomial logistic regression model was used to test which factors had an association with treatment outcome in the TB-HIV population. The reference utilized to test associations was a favorable treatment outcome. The following variables were statistically associated with an unfavorable treatment outcome, alcohol consumption (reference: no for alcohol consumption), illicit drug use (reference: no for illicit drug use), non-white (reference: white ethnicity), prior TB (reference: new TB diagnosis cases), and non-PTB (reference: PTB). While age lower than 40 (reference: age higher than 56), age between 40 and 56 (reference: age higher than 56) and male (reference: female sex) increased the likelihood of progressing into a favorable outcome. Diabetes (reference: non-diabetes), tobacco use (reference: no for tobacco use), suspected X-ray (reference: normal x-ray), and less than HS diploma (reference: more than HS diploma) were all not statistically significant. TB, tuberculosis; HIV, human immunodeficiency virus; PTB, pulmonary tuberculosis; HS, high school; Non-PTB, extrapulmonary tuberculosis.

Finally, we performed a logistic regression analysis which demonstrated that no independent associations were found between occurrence of unfavorable treatment outcome and the presence of DM [adjusted Odds Ratio (aOR): 0.97, 95% Confidence Interval (CI): 0.83–1.12, *p* = 0.718] (Figure 2). On the other hand, certain variables did have an association with unfavorable outcomes, including: history of prior TB (aOR: 1.82, 95% CI: 1.72–1.93, *p* < 0.001), illicit drug use (aOR: 1.53, 95% CI: 1.42–1.64, *p* < 0.001), alcohol consumption (aOR: 1.26, 95% CI: 1.17–1.35, *p* < 0.001); non-white ethnicity (aOR: 1.18, 95% CI: 1.12–1.25, *p* < 0.001), and non-PTB (aOR: 1.08, 95% CI: 1.02–1.14, *p* = 0.005). While age lower than 40 years (aOR: 0.83, 95% CI: 0.75–0.93, *p* = 0.001), age between 40 and 56 years (aOR: 0.83, 95% CI: 0.75–0.92, *p* = 0.001) and male sex (aOR: 0.80, 95% CI: 0.76–0.85, *p* < 0.001) were associated with favorable outcomes (Figure 2).

**Figure 3:**
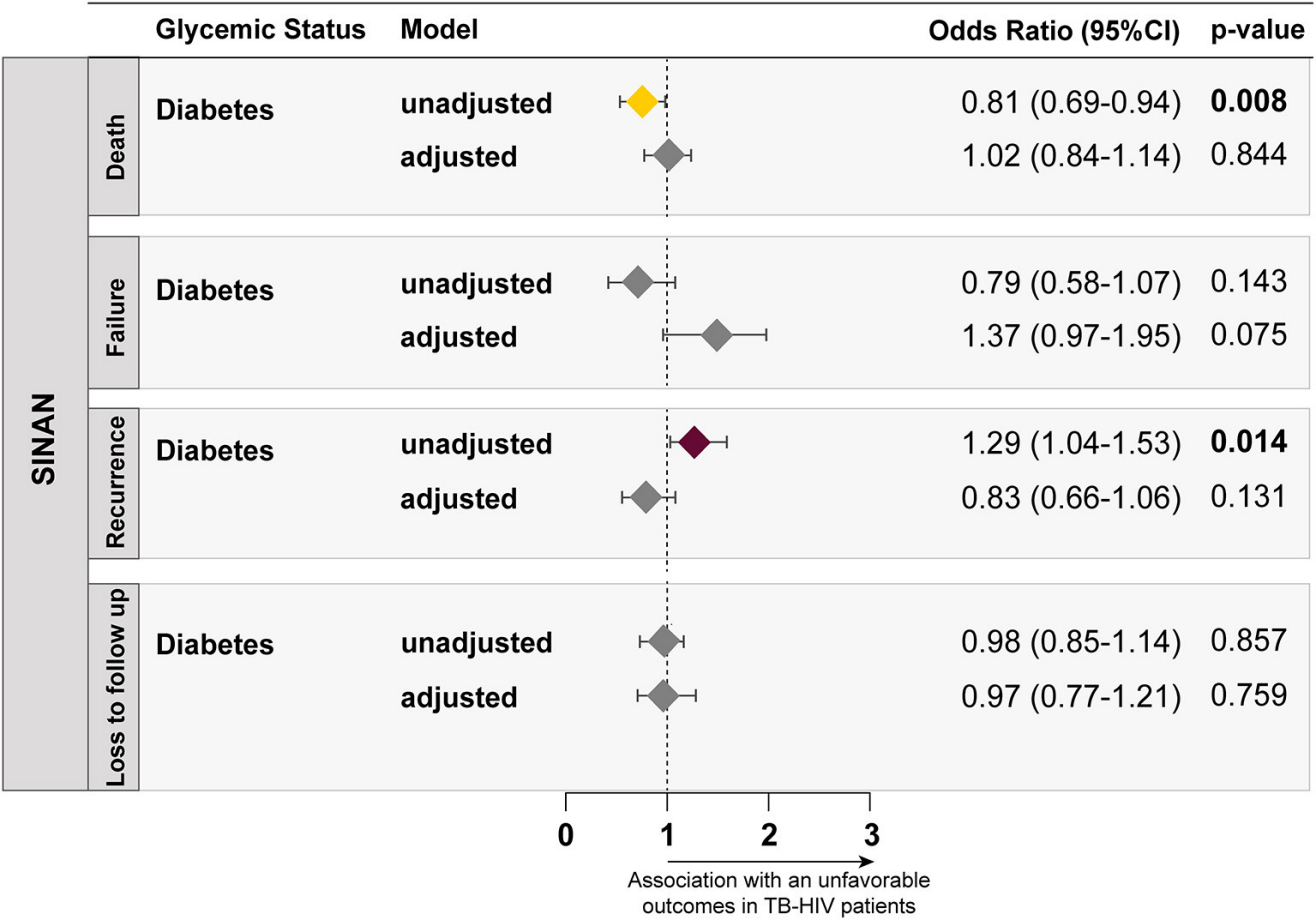
Four multivariable logistic regression models, utilizing the ENTER method, testing associations between the presence of DM (reference: no DM) and different subcategories of unfavorable outcomes, including death (reference: clinical or bacteriological cure), treatment failure (reference: clinical or bacteriological cure), TB recurrence (reference: clinical or bacteriological cure) and loss to follow up (reference: clinical or bacteriological cure), were performed and plotted. All the variables included in the models can be found on Supplementary Table 3.

Moreover, four additional binomial regressions were conducted aiming to evaluate the relationship between DM and each specific unfavorable outcome utilized in this study. In all cases, no statistical association was identified between these variables, the following were the results regarding presence of DM: model 1: death (aOR: 1.02, 95% CI: 0.84–1.14, *p* = 0.844), model 2: treatment failure (aOR: 1.37, 95% CI: 0.97–1.95, *p* = 0.075), model 3: recurrence (aOR: 0.83, 95% CI: 0.66–1.06, *p* = 0.131) and model 4: loss to follow up (aOR: 0.97, 95% CI: 0.77–1.06, *p* = 0.759) (Figure 3). The full results, with all covariates included the analysis, can be found in Supplementary Table 3.

Additionally, we also compared the frequency of TB drug resistance in the DM and non-DM groups (Supplementary Figure 1). We observed no statistically significant differences in the occurrence of any drug resistance (Supplementary Figure 1A), any multidrug resistance (Supplementary Figure 1B), or any distinct profile of monodrug resistance (Supplementary Figure 1C). In fact, the number of cases with multidrug resistance was very small among DM cases (Supplementary Figure 1D). Finally, we designed a Sankey diagram to illustrate association between DM, drug sensitivity and anti-TB treatment outcomes (Supplementary Figure 2). The results demonstrated that the frequencies of drug resistant case were so low that precluded observation of substantial association with treatment outcomes. Therefore, we could not include drug-resistance as a variable in the multivariable analysis due to the unfortunate low rates of documented drug resistance in the clinical group of interest.

## Discussion

Tuberculosis is an airborne communicable disease that caused the death of ~1.3 million people in 2020 (7). However, by addressing TB determinants such as DM, alcoholism, under nutrition, HIV infection, poverty and smoking tobacco it is possible to lower the mortality of this disease (7). There are many studies which evaluated the adverse effects that HIV and DM individually have on ATT outcomes, however current literature is scarce regarding coexistence with these three health conditions. To our knowledge, this is the first study that has investigated the effects of DM on the treatment outcomes of TB-HIV patients, using a nationwide database. This paper addressed the question of whether there are determinant factors associated with an unfavorable ATT outcome in TB-HIV-DM patients, while also analyzing results found in populations of TB-HIV patients without DM.

We noticed that the same variables were found to have higher frequency rates in those with an unfavorable treatment outcome in all populations, regardless of the DM status. Those include characteristics such as being of the non-white ethnicity, the adoption of certain consumption habits, as well as presence of prior TB. Regarding ethnicity rates, this may be explained by social inequality in Brazil, as for historical reasons, in this country, white individuals have on average a better socioeconomic status and, therefore, may have greater odds to adhere to therapy (14).

Concerning drug consumption habits, our data showed that patients who drink alcohol, use illicit drugs, and smoke tobacco have a higher presentation in unfavorable outcomes groups regardless of the DM status. This can also be explained by the fact that these life habits are risk factors for several diseases in addition to negatively influencing DM patients (15–18). Excessive use of alcohol and tobacco use have been shown to be associated with more deadly and contagious forms of TB disease, including higher rates of smear positive results, drug toxicity, disease reactivation, as well as with lower economic status and lower adherence to therapy (19, 20).

Patients with history of prior TB were found here to exhibit higher percentages of unfavorable outcomes, while new TB cases were more frequently present in favorable outcome groups. We hypothesize that this can be explained due to potentially patients with history of prior TB may have a lower adherence to repeat treatment or present multidrug resistance (21, 22).

Furthermore, our results show that the presence of DM was not statistically associated with an overall unfavorable ATT outcome in TB-HIV patients. Moreover, DM did not individually impact any of the subcategories that composed an unfavorable ATT outcome in this database, including death, treatment failure TB recurrence and loss to follow up. In addition, we demonstrated that certain determinant factors which are associated with impacts on ATT outcomes had an extremely similar presentation between all population groups. Both findings differ from the results found in other studies analyzing TB-DM co-infection, the majority of which reported that DM as a comorbidity that can worsen clinical symptoms, increase the likelihood of developing active TB and of disease recurrence, as well as negatively impact a subject's treatment outcome (23–25). Additionally, the co-association between DM and HIV has also been reported to worsen the course of both pathologies, increasing risk of death by several times in some studies (26, 27). However, there are also studies which have demonstrated that the presence of DM does not increase risk of developing active TB among specifically PLWH, which differs from the three-fold increase that it has on patients who are only infected by TB (23, 26). All of this seems to imply that the presence of DM as another comorbidity does not seem to have a significant impact the outcome of TB-HIV.

An important aspect to be considered when one is evaluating determinants of anti-TB treatment outcomes is drug resistance, which directly impacts adequate therapeutic response. Herein we failed to observe a significant association between the profile of anti-TB drug resistance and treatment outcomes, likely due to the fact that in this national TB dataset, frequencies of distinct drug-resistance profiles were low in the group of study participants with DM. Our results indicate that drug resistance does not seem to be a substantial problem in the context of TB-HIV-DM, albeit such findings require further validation in other epidemiological settings to confirm this lack of association. In fact, in our dataset, there was a considerable high frequency of individuals who did not have recorded the results of drug sensitivity tests. It is possible that increased drug sensitivity screening could result in higher numbers of drug-resistant TB, and, thus, future studies are necessary to confirm this hypothesis.

Our study had some limitations, including that several variables that had significant levels of under-reporting as well as missing numbers. Although, variables with extremely high rates of missing cases were excluded from the study, including data regarding drug-resistance and cash transfer programs. Moreover, the SINAN-TB database does not possess biochemical and pathological data of the reported cases. Thus, our analysis was limited to statistical inferences using clinical and epidemiological characteristics. In addition, all data regarding DM was self-reported, thus values regarding fasting blood glucose levels and hemoglobin A1c (HbA1c) levels were not documented, also there was no differentiation between if patients had either the type 1 or type 2 form of this disease. Additionally, as we did not link the SINAN-TB and SINAN-HIV databases, we did not have access to the ART use and when it was initiated during ATT. Also, we were uncertain if all data was collected uniformly throughout the regions of the country. Nonetheless, even with these limitations, a study like this is important since it was, to our knowledge, the first to analyze specifically a large amount of data about of TB-HIV-DM population in the Brazilian literature, when concerning significant factors that were associated with progression to unfavorable treatment outcomes.

Our results demonstrate that, the DM does not seem have a significant impact on their treatment outcome in our sample. Additionally, variables such as certain consumption habits, prior TB and non-white ethnicity had a similar presentation in unfavorable ATT outcome groups of both TB-HIV and TB-HIV-DM patients. Therefore, this study highlights the importance of also investing time and resources among TB-HIV infected patients in controlling the use of alcohol, tobacco and other drugs, as well as the significance of prior TB cases and the social characteristics of TB patients, since, in contrast to DM, all of these had a large presence in groups with a worse prognosis. This type of strategy can minimize the occurrence of unfavorable outcomes of anti-TB treatment and improve this population's quality of life.

## Data availability statement

The raw data supporting the conclusions of this article will be made available by the authors, without undue reservation.

## Ethics statement

Ethical review and approval was not required for the study on human participants in accordance with the local legislation and institutional requirements. Written informed consent for participation was not required for this study in accordance with the national legislation and the institutional requirements.

## Author contributions

KV-S, BB-D, VN, RM, MA-P, and BA contributed to conception and design of the study, processed and analyzed the data, worked on data visualization, and wrote the first draft of the manuscript. KV-S, BB-D, VN, RM, MR, AQ, MA-P, and BA performed the data curation. MA, MC-S, AK, and TS revised and contributed to the structuring of the article. TS, MA-P, and BA supervised the research. All authors contributed to manuscript revision, read, and approved the submitted version of the manuscript.

## Funding

This study was supported by the National Institutes of Health (NIH U01 AI069923 and NIAID R01 P30AI110527-03), CCASAnet, RePORT-Brazil, Tennessee Center for AIDS Research (TNCFAR), BB-D, RM, and MA-P received a research fellowship from the Coordenação de Aperfeiçoamento de Pessoal de Nível Superior (CAPES) (Finance Code: 001). KV-S received a fellowship from the Fundação de Amparo à Pesquisa da Bahia (FAPESB). The work of BA is supported by the Intramural Research Program of the Oswaldo Cruz Foundation (FIOCRUZ) and the National Council for Scientific and Technological Development (CNPq), Brazil.

## Conflict of interest

The authors declare that the research was conducted in the absence of any commercial or financial relationships that could be construed as a potential conflict of interest.

## Publisher's note

All claims expressed in this article are solely those of the authors and do not necessarily represent those of their affiliated organizations, or those of the publisher, the editors and the reviewers. Any product that may be evaluated in this article, or claim that may be made by its manufacturer, is not guaranteed or endorsed by the publisher.
