# Supplementary material for: Tuberculosis treatment outcomes of diabetic and non-diabetic TB/HIV co-infected patients: A nationwide observational study in Brazil
Source: Front Med (Lausanne). 2022 Sep 16;9:972145. doi: 10.3389/fmed.2022.972145 (PMC9523014; doi:10.3389/fmed.2022.972145)
Supplement: Supplementary file 1 [file Data_Sheet_1.PDF]

Supplementary Material

SUPPLEMENTARY MATERIAL

Content

1. Supplementary Table 1

2. Supplementary Table 2

3. Supplementary Table 3

4. Supplementary Figure 1

5. Supplementary Figure 2

Supplementary Tables:

Supplementary Table 1. Definitions of tuberculosis treatment outcomes

Type of Outcome Treatment	Characterization of ATT outcome	SINAN Definitions[a]
**Favorable**	Cure	Defined a pulmonary TB patient, which initially had a positive sputum smear or Xpert result, and then exhibits at least 2 negatives sputum smear during treatment, being one during the follow-up fase and the other at the end of treatment.
**Unfavorable**	Death	Defined as the knowledge of a patient's death during treatment
	Treatment Failure	When a positive sputum smear results remains until the end of treatment. Additionally, patients who at the start ATT are strongly positive (++ or +++) and persist at this level until the end of the fourth month or those with an initial positivity followed by negativity and return to positivity for 2 consecutive months from the fourth month of ATT.
	Loss to Follow Up	Cases which the patient has taken more than 30 consecutive days to return to a treatment unit after the expected return date. In cases of supervised treatment, it is considered 30 days from the last date of taking the drug.
	Recurrence	A patient who was cured of active TB through past ATT and now presents the with disease again, regardless of the time since the previous treatment.
	Transferred	Defined as a case where the patient was transferred to another health unit.

Table note: [a]Related from the Manual of Recommendations for the Control of TB of Brazil. Abbreviations:SINAN: Sistema de Informação de Agravos de Notificação. TB: Tuberculosis, ATT: Anti-Tuberculosis Therapy

Supplementary Table 2. Characterization of Brazilian TB-HIV-DM patients

Characteristics	All (n=31070)	DM (n=999)	Non-DM (n=30071)	p-value	pδ-value
**Male, *n* (%):**	21801 (70.2)	690 (69.1)	21111 (70.2)	0.462	0.3
**Age Categories, *n* (%):**				**<0.001**	**0 (Δ=0.57)**
Lower than 40	16929 (54.5)	239 (23.9)	16690 (55.5)		
Between 40-56	11469 (36.9)	489 (48.9)	10980 (36.5)		
Higher than 56	2672 (8.60)	271 (27.1)	2401 (7.98)		
**Non-white, *n* (%):**	20931 (67.4)	674 (67.5)	20257 (67.4)	0.973	0.51
**Educational Levels, *n* (%):**				**0.008**	**0(Δ=0.03)**
Less than HS Diploma	3069 (10.8)	121 (13.5)	2948 (10.7)		
More HS diploma	25396 (89.2)	775 (86.5)	24621 (89.3)		
**HIV, *n (%):***	31070 (100)	999 (100)	30071 (100)	ns	ns
**Alcohol consumption, *n* (%):**	6733 (22.2)	277 (29.4)	6456 (21.9)	**<0.001**	1
**Illicit Drug Use, *n* (%):**	5987 (20.4)	172 (19.3)	5815 (20.5)	0.434	0.06
**Tobacco use, *n* (%):**	6474 (21.9)	262 (29.0)	6212 (21.7)	**<0.001**	1
**Smear Positive, *n* (%):**	10850 (96.4)	410 (97.9)	10440 (96.3)	0.126	1
**Culture Positive, *n* (%):**	5628 (58.1)	198 (59.8)	5430 (58.1)	0.565	0.84
**Suspect Chest x-ray for TB, *n* (%):**	22482 (73.7)	741 (74.9)	21741 (73.7)	0.696	0.83
**Prior TB, *n* (%):**	8780 (28.3)	276 (27.6)	8504 (28.3)	0.678	0.38
**Type of TB, *n* (%):**				**<0.001**	1
PTB	20925 (67.3)	747 (74.8)	20178 (67.1)		
Non-PTB	10145 (32.7)	252 (25.2)	9893 (32.9)		

Table note: Data represents frequency (%). Categorical variables were compared using Pearson's chi-square test.

Definition of alcohol consumption: Past or current any consumption of alcohol. *Definition of tobacco use*: Past or current smoking of tobacco. *Definition of illicit drug use:* Past or current illicit drug use (marijuana, cocaine, heroin or crack). *Definition of non-white: The following self-reported races: Asian, Black, Pardo and Indigenous. Definition of type of TB*: Clinical form of TB regarding the disease's location. *Definition of prior-TB:* Previous TB history. **Abbreviations:** TB: tuberculosis, PTB: Pulmonary Tuberculosis, Non-PTB: Extrapulmonary and Disseminated Tuberculosis, ns: Not Significant, pδ-value: second-generation p-value and Δ = delta-gap.

Supplementary Table 3. Adjusted odds ratio for specific unfavorable treatment outcomes in TB-HIV patients

Parameter	AOR (95% CI) for Death	AOR (95% CI) for Failure	AOR (95% CI) for Relapse	AOR (95% CI) for Loss to Follow Up
**Diabetes Mellitus**	1.02 (0.84-1.23)	1.37 (0.97-1.95)	0.83 (0.66-1.06)	0.97 (0.77-1.21)
**Tobacco use**	0.92 (0.84-1.01)	1.57 (1.33-1.86)	1.02 (0.92-1.12)	1.18 (1.06-1.31)
**Alcohol Consumption**	1.33 (1.21-1.46)	1.20 (1.01-1.44)	1.26 (1.14-1.39)	1.19 (1.07-1.32)
**Illicit Drug Use**	1.18 (1.06-1.31)	1.55 (1.30-1.85)	2.15 (1.95-2.36)	1.30 (1.17-1.45)
**Male**	0.91 (0.84-0.97)	0.72 (0.63-0.83)	0.72 (0.67-0.78)	0.80 (0.74-0.87)
**Suspected X-Ray**	1.08 (0.99-1.17)	1.30 (1.10-1.53)	0.99 (0.91-1.08)	0.88 (0.80-0.96)
**Non-white**	1.26 (1.17-1.36)	0.92 (0.80-1.05)	1.31 (1.21-1.42)	1.02 (0.94-1.10)
**Prior TB**	1.38 (1.27-1.49)	2.54 (2.22-2.92)	2.53 (2.35-2.73)	1.62 (1.49-1.77)
**Less than HS Diploma**	0.91 (0.82-1.02)	0.82 (0.65-1.02)	1.07 (0.96-1.20)	0.88 (0.78-1.00)
**Non-PTB**	1.21 (1.12-1.30)	1.45 (1.26-1.68)	0.80 (0.74-0.87)	1.18 (1.09-1.29)
**Age Lower than 40**	0.50 (0.44-0.56)	1.16 (0.86-1.56)	1.94 (1.61-2.32)	0.85 (0.73-0.98)
**Age between 40-56**	0.65 (0.58-0.74)	1.11 (0.82-1.51)	1.36 (1.13-1.64)	0.86 (0.74-1.00)

Table note: Data represents adjusted odds ratio (AOR) and 95% confidence intervals (CI). Categorical variables were analyzed utilizing four multinomial logistic regression models, with each analysis having a different treatment outcome as dependent variable. The reference utilized to test associations in all models was TB clinical or bacteriological cure. *Definition of alcohol consumption:* Past or current any consumption of alcohol. *Definition of tobacco use:* Past or current smoking of tobacco. *Definition of illicit drug use:* Past or current illicit drug use (marijuana, cocaine, heroin or crack) *Definition of non-white:* The following self-reported races: Asian, Black, Pardo and Indigenous. *Definition of type of TB:* Clinical form of TB regarding the disease's location. *Definition of prior-TB:* Previous TB history. **Abbreviations:** TB: tuberculosis, PTB: Pulmonary Tuberculosis, Non-PTB: Extrapulmonary and Disseminated Tuberculosis, HIV: Human Immunodeficiency Virus, HS: High School.

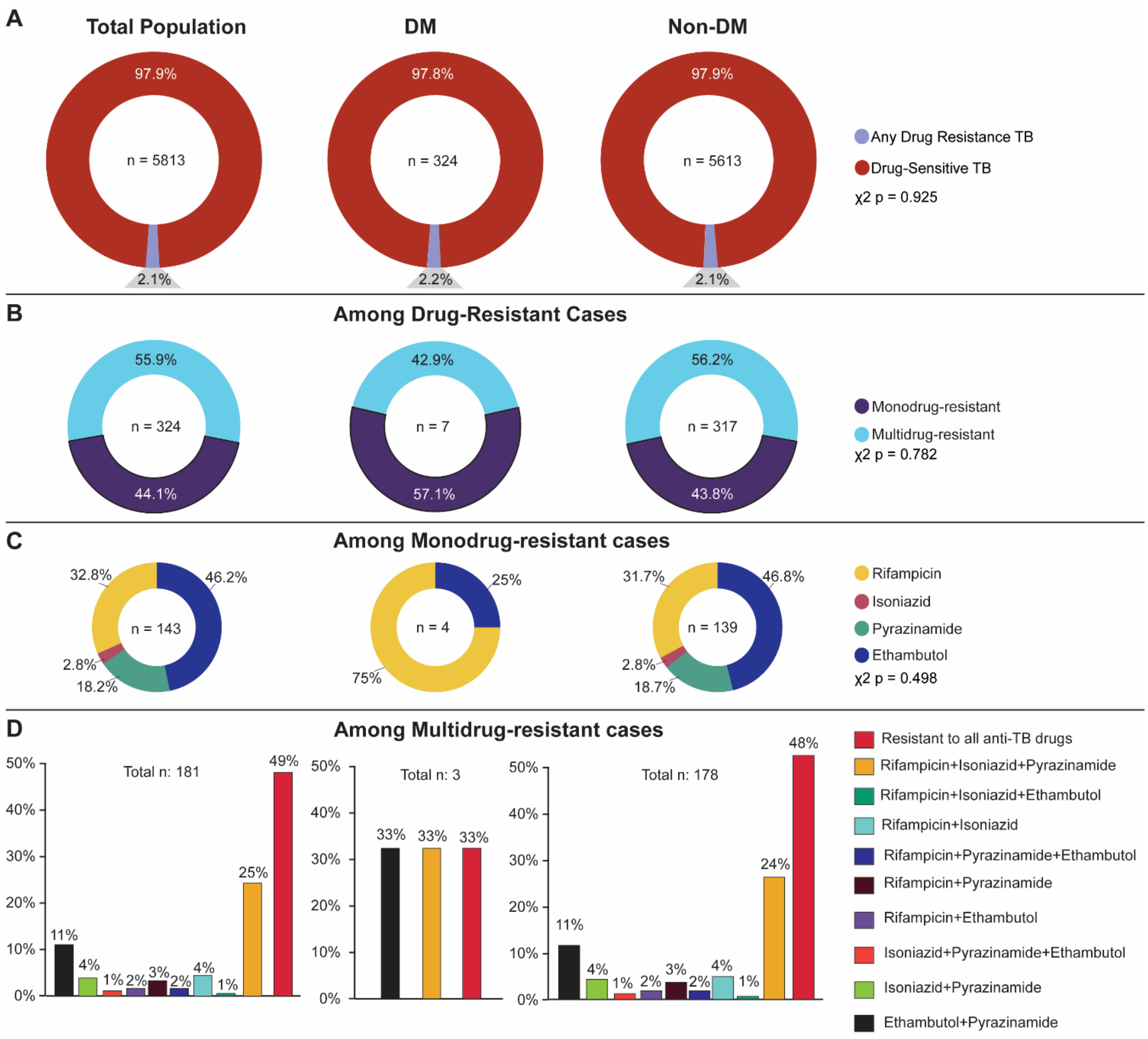

Supplementary Figure 1. Tuberculosis treatment outcomes in the TB-HIV population stratified according to DM and TB Drug Sensitivity in the Brazilian national TB notification system. (A) Frequencies of study participants presenting with DST or those who presented any anti-TB drug resistance. (B) Frequencies of mono or multidrug-resistant TB among drug-resistant cases. (C) Frequencies of distinct types of monodrug-resistant TB. (D) Frequencies of distinct types of multidrug-resistant TB. Frequencies were compared between the groups of participants with and without DM using the Pearson's chi-square test.

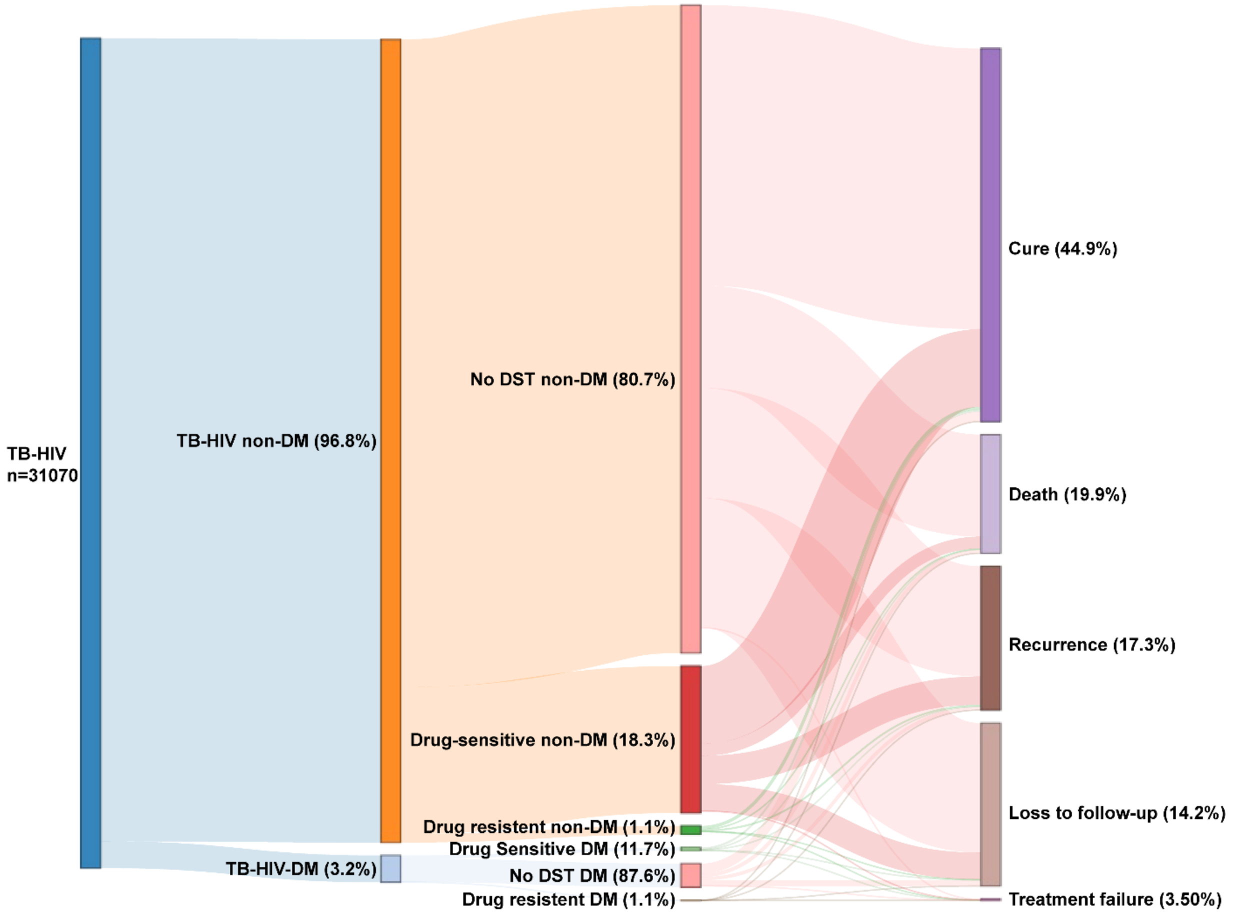

Supplementary Figure 2. Frequencies of TB treatment outcomes in subpopulations of TB-HIV patients with and without DM according to results of anti-mycobacterial drug sensitivity tests. Sankey plot shows the stratifications of study participants according to occurrence of DM, drug sensitivity results and anti-TB treatment outcomes. Abrreviations: TB: tuberculosis; DM: diabetes melittus; DST: drug sensitivity test.
